# Modular polyketide synthase ketosynthases collaborate with upstream dehydratases to install double bonds†

**DOI:** 10.1039/d4cc03034f

**Published:** 2024-07-19

**Authors:** Katherine A. Ray, Nisha Saif, Adrian T. Keatinge-Clay

**Affiliations:** a Department of Molecular Biosciences, The University of Texas at Austin 100 E. 24th St. Austin TX 78712 USA adriankc@utexas.edu

## Abstract

A VMYH motif was determined to help ketosynthases in polyketide assembly lines select α,β-unsaturated intermediates from an equilibrium mediated by an upstream dehydratase. Alterations of this motif decreased ketosynthase selectivity within a model tetraketide synthase, most significantly when replaced by the TNGQ motif of ketosynthases that accept d-β-hydroxy intermediates.

## Abstract 2

A VMYH motif was determined to help ketosynthases in polyketide assembly lines select α,β-unsaturated intermediates from an equilibrium mediated by an upstream dehydratase.

## Graphical abstract

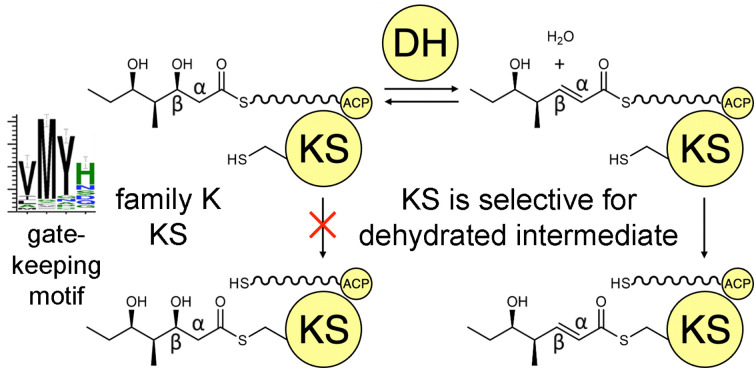

Modular polyketide synthases (PKSs) are enzymatic assembly lines in which tens to hundreds of domains collaborate to synthesize bioactive natural products like the antibacterial pikromycin and the antifungal amphotericin (Fig. 1).^1,2^ Sets of domains that extend and modify the growing polyketide intermediate are known as modules.^3^ The modules of *cis*-acyltransferase (*cis*-AT) assembly lines like the pikromycin and amphotericin PKSs are minimally composed of an AT domain that selects an extender unit from an acyl-CoA, an acyl carrier protein (ACP) domain that acquires this extender unit as well as the growing polyketide, and a ketosynthase (KS) domain that catalyzes carbon–carbon bond formation between the two.^4,5^ A module may also possess a ketoreductase (KR) that reduces the β-keto group formed by KS, a dehydratase (DH) that eliminates the β-hydroxy group formed by KR, and an enoylreductase (ER) that reduces the α,β-unsaturated intermediate formed by DH. These processing enzymes are responsible for most of the diversity observed in complex polyketides.

**Fig. 1:**
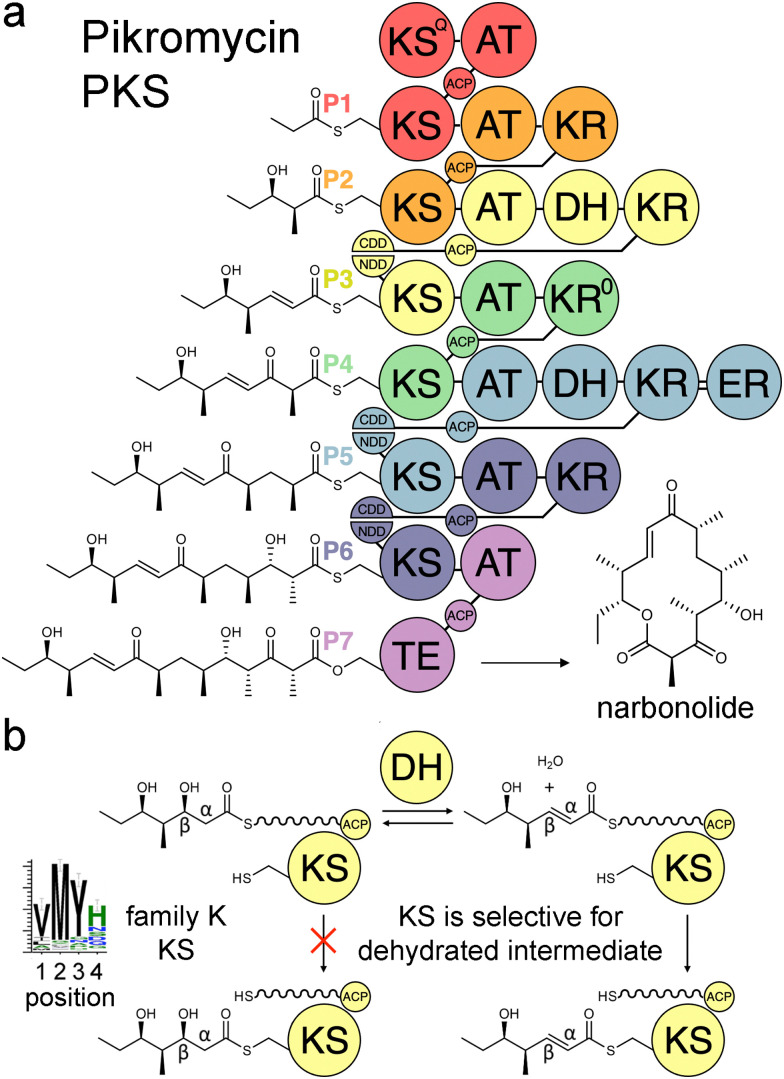
Double-bond formation in the pikromycin PKS. (a) Modules are colored according to the updated boundary. (b) DH mediates an equilibrium between hydrated and dehydrated intermediates. The KS from the third module, PikKS3, is hypothesized to gatekeep for the dehydrated intermediate using a VMYH motif. CDD/NDD, docking motifs; KS^Q^, priming KS; KR^0^, epimerase; TE, thioesterase.

Over the last three decades attempts to recombine modules into PKSs that synthesize novel products have seldom been rewarded.^6,7^ Most engineering has been performed using the traditional definition of the module in which KSs are grouped with the processing enzymes downstream of them. However, the recent realization that KSs evolutionarily co-migrate with the processing enzymes upstream of them has led to an updated definition of the module.^8–10^ Engineering with updated modules has been shown to be superior to engineering with traditional modules.^11–15^ One reason for this may be that KS substrate tunnels are complementary to the intermediates they naturally accept, at least at the α- and β-positions, so fewer incompatibilities are encountered when KSs and the processing enzymes naturally upstream of them that set the chemistries at the α- and β-positions are kept together. A bioinformatics study of 739 KSs from 92 characterized, actinomycete PKSs identified motifs that may help KSs gatekeep for intermediates processed by upstream enzymes.^16^ KSs were sorted into families A–P based on the chemistries at the α- and β-positions of their substrates, and 32 residues in their substrate tunnels were investigated. Residues at positions 1–4 on the dimer interface loop were identified to be among the most relevant to gatekeeping.^17^ The strongest motif observed in these positions is the VMYH motif conserved in family K, L, and M KSs that accept an α,β-unsaturated intermediate from an upstream DH.^8,16^ As DH can only establish an equilibrium between the β-hydroxy intermediate and its dehydrated product,^18^ a selection for the dehydrated intermediate must be performed by a downstream enzyme such as KS. The methionine in position 2 of the VMYH motif is appropriately located to make hydrophobic interactions with the double bond of an α,β-unsaturated intermediate and sterically exclude the β-hydroxy group of a d-β-hydroxy intermediate.^16^ The TNGQ motif conserved in family F, I, and J KSs that accept a d-β-hydroxy intermediate is also quite strong. The asparagine in position 2 has been proposed to orient the side-chain carbonyl of the conserved glutamine at position 31 such that it can form a hydrogen bond with the d-β-hydroxy group of the processed intermediate but repel the β-keto group of the unprocessed intermediate.^16^

In this study, the VMYH motif of the KS from the 3rd module of the pikromycin synthase, PikKS3, was probed by mutagenesis to investigate whether it helps gatekeep for the α,β-unsaturated intermediate generated by the upstream DH, PikDH3 (Fig. 2). Mutagenesis was performed on P1-P2-P3-P7, a tetraketide synthase our lab recently constructed from the 1st, 2nd, 3rd, and 7th updated modules of the pikromycin synthase (P1, P2, P3, and P7) (Tables S1 and S2, ESI†).^15^ This engineered PKS produces tetraketide olefin 1 ([M + H]^+^: 171.1380 Da) and a much smaller quantity of tetraketide lactone 2 ([M + H]^+^: 215.1278 Da). It also produces a small quantity of a shunt product, triketide lactone 3 ([M + H]^+^: 171.1016 Da). As the pathway to 3 most likely involves module-skipping,^4,19^ its production does not provide insight into the gatekeeping mechanisms of the skipped third module, P3. If mutations to the VMYH motif alter the ability of PikKS3 to select against the β-hydroxy intermediate, synthases containing them may produce a lower 1 : 2 ratio. As the methionine and tyrosine are close to where the α- and β-positions of the polyketide intermediate would be during transacylation to PikKS3, each was mutated to alanine to create the VAYH, VMAH, and VAAH variants. Residues from the VMYH motif were also incrementally replaced with residues from TNGQ motif conserved in family F, I, and J KSs that are acylated by β-d-hydroxyacyl intermediates, generating the VNYH, VNGH, TNGH, and TNGQ variants. Because swapping this 4-residue motif might not be sufficient to change PikKS3 specificity, the entire PikKS3 domain was also swapped with AmpKS15, a family F KS from the 15th module of the amphotericin synthase, generating the AmpKS15 variant.

**Fig. 2:**
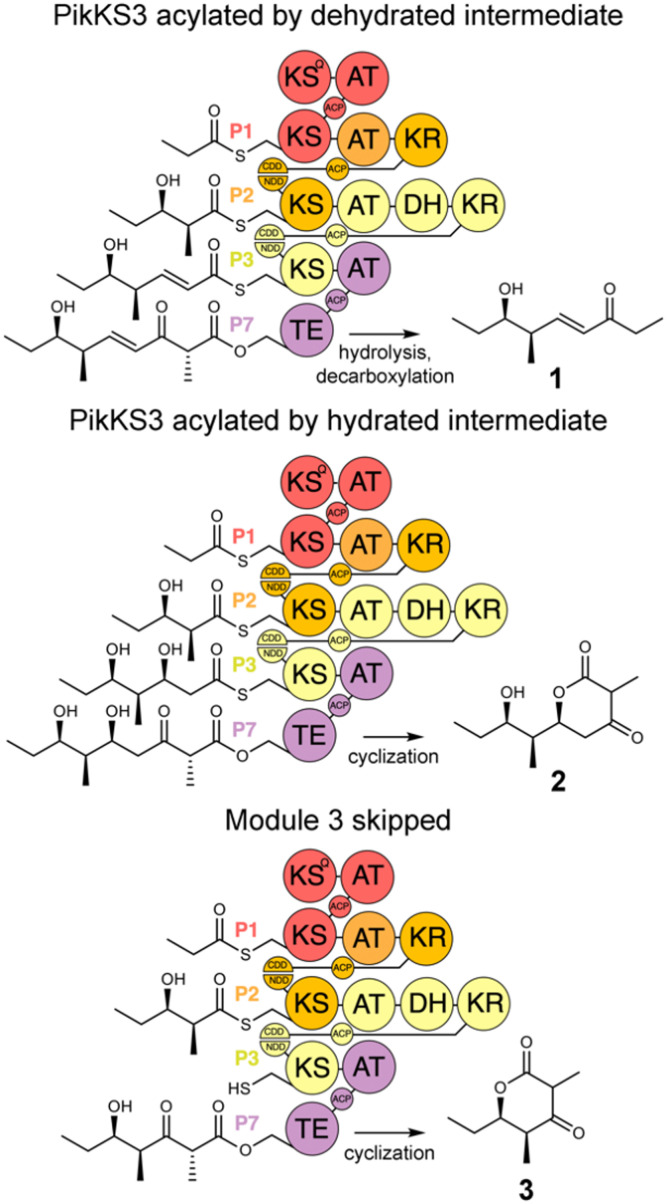
Polyketide production by P1-P2-P3-P7 and variants. P1-P2-P3-P7, engineered from the updated modules of the pikromycin synthase, synthesizes a β-ketoacid that spontaneously decarboxylates to the tetraketide olefin 1. Tetraketide lactone 2 is generated when PikKS3 is acylated by the hydrated intermediate. Triketide lactone 3 is generated when the third module, P3, is skipped.

Expression plasmids encoding P1-P2-P3-P7 and its variants were transformed into *E. coli* K207-3, an engineered strain that activates ACP domains through phosphopantetheinylation and generates (2*S*)-methylmalonyl-CoA extender units from propionate supplied to the media.^20^ Cells were grown in shake flasks containing polyketide-production media, and production was initiated through the addition of 0.1 mM isopropyl β-d-1-thiogalactopyranoside and 20 mM sodium propionate. After cultures were incubated for 7 d at 19 °C, 500 μL of culture media was acidified with 10 μL concentrated aqueous hydrochloric acid, extracted with ethyl acetate, and dried *in vacuo*. The extract was resuspended in 500 μL of 1 : 1 (v/v) methanol/water and analyzed by liquid chromatography/mass spectrometry (LC/MS) for polyketide production (Fig. S1–S7, ESI†). The levels of 1, 2, and 3 produced in the culture (expressed in μM) were measured using standard curves (Fig. 3 and Fig. S8–S11, data file 1, ESI†).

**Fig. 3:**
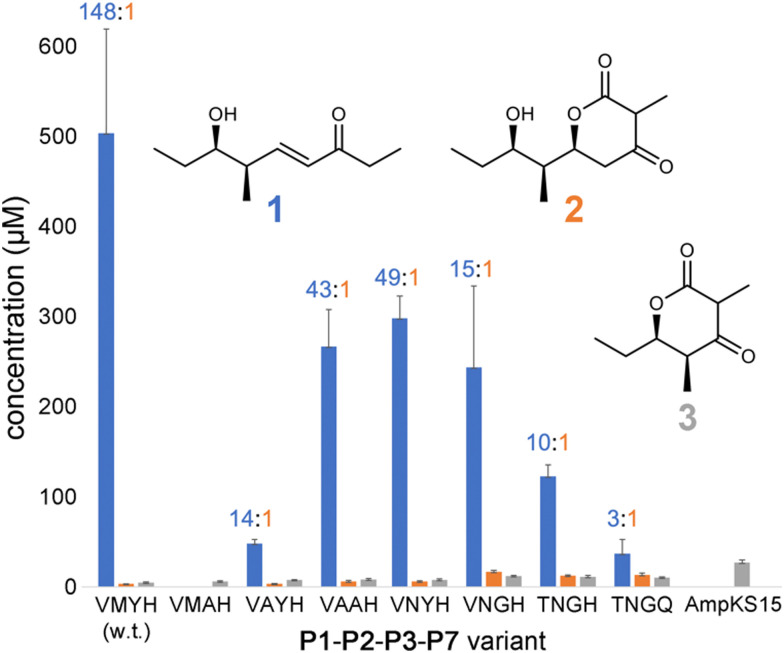
Titers of 1–3 produced by P1-P2-P3-P7 and variants. Titers of 1 were quantified from peak areas of LC/MS extracted ion chromatograms using a standard curve, while 2 and 3 were quantified by UV absorption. The 1 : 2 ratio is indicated for each tetraketide-producing variant.

P1-P2-P3-P7 produces a 1 : 2 ratio of 148, indicating that while wild-type PikKS3 is very selective, its gatekeeping activity is not perfect. Each of the mutations made to the VMYH motif resulted in a lower 1 : 2 ratio. Large changes in the production of 1 were observed when either the methionine or tyrosine was mutated. An order of magnitude decrease in the production of 1 was observed for the VAYH variant (1 : 2 = 14), and no production of 1 or 2 was observed from the VMAH variant. The variants possessing incremental mutations converting VMYH to TNGQ also produced lower 1 : 2 ratios: 49 for the VNYH variant, 15 for the VNGH variant, 10 for the TNGH variant, and 3 for the TNGQ variant. That the TNGQ variant still yields more 1 than 2 is not unexpected. Family F, I, and J KSs, which contain the TNGQ motif, are in modules that do not dehydrate intermediates and thus evolved to select against β-keto intermediates rather than α,β-unsaturated intermediates. The VMAH and AmpKS15 variants did not produce 1 or 2 but did produce shunt product 3, like the other variants.

To learn more about the interactions between residues at positions 1–4 of PikKS3 and the d-β-hydroxy and α,β-unsaturated intermediates, models of PikKS3 and its variants bound to these intermediates were generated (Fig. 4 and Fig. S12, ESI†). PikKS3 structures were predicted with AlphaFold 2.0,^21^ while coordinate and restraint files for the methyl thioester analogs of both intermediates were generated by the program Sketcher.^22^ The program Coot was used to position the intermediates as in experimentally-determined acyl-KS structures (PDBs: 2BUI, 2GFY, 2IX4, 6ROP, 7UK4)^23–29^ where the hydrogen bond distance between the thioester oxygen and the amide nitrogen of position 32 ranges from 2.7 to 3.1 Å, the N–C_α_–C_β_–S torsion angle of the reactive cysteine ranges from −59° to −96°, and the O–C–C_α_–C_β_ torsion angle of the acyl chain ranges from −48° to −88° (applicable to the β-hydroxy intermediate, 0° was used for the enoyl intermediate, Table S3, ESI†). In PikKS3, the position 2 methionine interacts hydrophobically with the α,β-double bond and sterically repels β-substituents. Through hydrogen bonds with the side chains of a glutamine conserved at position 31 and a semi-conserved threonine at position 22, the position 3 tyrosine may stabilize the conformation of the dimer interface loop and help orient the position 2 methionine. In family F KSs and the TNGQ variant of PikKS3, the dimer interface loop is predicted to be in a different conformation from that of a VMYH-containing loop. In this conformation, the position 2 asparagine can form a hydrogen bond with the position 31 glutamine such that the glutamine side chain carbonyl is poised to accept a hydrogen bond from the d-β-hydroxy group of a hydrated intermediate and repel the β-keto group of an unreduced intermediate.

**Fig. 4:**
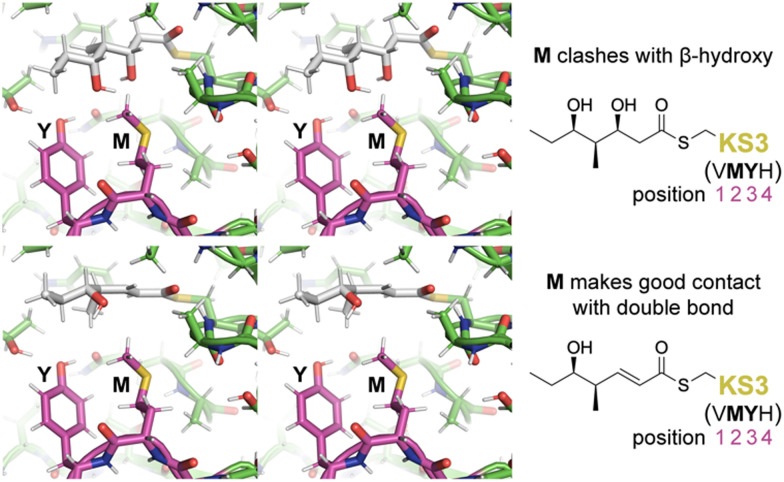
Gatekeeping by the VMYH motif. Stereodiagrams show the hydrated and dehydrated intermediates modeled in PikKS3 according to observed acyl-KS structures. While the methionine clashes with the β-hydroxyl group of the hydrated intermediate, it makes good hydrophobic contact with the dehydrated intermediate near the double bond (see also Fig. S12, ESI†). The dimer interface loop containing the position 1–4 gatekeeping residues is shown in magenta.

In this work we demonstrated that PikKS3 collaborates with the upstream processing enzyme, PikDH3, to select the dehydrated over the hydrated intermediate from the PikDH3-mediated equilibrium. Its position 2 methionine plays the major role in selecting against the hydrated intermediate. The residue in position 2 has been observed to mediate selectivity in other KS families. An asparagine conserved in this position in family D KSs downstream of C2-type KRs (*e.g.*, KR^0^ in P4, Fig. 1) that epimerize α-substituents is hypothesized to interact with the β-keto group of l-α-substituted, β-keto intermediates during the transacylation reaction.^16^ A tetraketide shunt product is observed when this asparagine is mutated to alanine in the 4th KS of the erythromycin PKS, indicating that the epimerized tetraketide intermediate cannot transacylate to this KS.^30^ Most KSs in the mycolactone, rapamycin, tautomycin, and tautomycetin PKSs possess an aromatic residue at position 2 hypothesized to enable the nonspecific binding of diverse polyketide intermediates.^16^ Indeed, increased substrate scope was observed for the family G KS in the 3rd module of the erythromycin PKS when the alanine in position 2 was replaced by a tryptophan.^31^

The recent successes utilizing updated modules (also referred to as “exchange units”) to engineer PKSs reflects the close collaboration of their domains.^11,12,14,15,32–36^ The partnership between upstream processing enzymes and downstream KSs that check for correct processing is most apparent in *trans*-AT assembly lines from the many KS^0^s that have evolutionarily lost their ability to form carbon–carbon bonds but have retained their ability to gatekeep.^5,10,37^ Recombining domains instead of modules to engineer hybrid synthases can result in the loss of interactions between domains that have evolutionarily comigrated.^8^ The inability of the AmpKS15 variant to generate tetraketide products may be due to an incompatibility between AmpKS15 and PikACP3 (Fig. S13, ESI†).^38^ While there are many details to learn about how KSs work together with upstream domains, they are what will allow access to the wealth of chemistries PKS modules have to offer in the production of designer polyketides.

K. A. R. and N. S. generated the expression plasmids for P1-P2-P3-P7 and its variants and performed the initial LC/MS analysis of the media extract from transformed cells. K. A. R. performed the LC/MS and UV analysis to determine production levels and high-resolution LC/MS and LC/MS/MS for characterization. A. T. K. performed the modeling. K. A. R. and A. T. K. prepared the manuscript.

This work was supported by the NIH (GM106112) and the Welch Foundation (F-1712).

## Data availability

The data supporting this article have been included as part of the ESI† and data file 1.

## Conflicts of interest

There are no conflicts of interest to declare.

## Supplementary Material

CC-060-D4CC03034F-s001

CC-060-D4CC03034F-s002
