# Supplementary material for: Modular polyketide synthase ketosynthases collaborate with upstream dehydratases to install double bonds
Source: Chem Commun (Camb). 2024 Jul 19;60(66):8712–5. doi: 10.1039/d4cc03034f (PMC11321453; doi:10.1039/d4cc03034f)
Supplement: CC-060-D4CC03034F-s002 [file CC-060-D4CC03034F-s002.pdf]

Electronic Supplementary Material (ESI) for ChemComm.
This journal is © The Royal Society of Chemistry 2024

SUPPLEMENTARY INFORMATION

Modular polyketide synthase ketosynthases collaborate with upstream dehydratases to install double bonds

Katherine A. Ray, Nisha Saif, Adrian T. Keatinge-Clay*

Department of Molecular Biosciences, The University of Texas at Austin, Austin, TX

*Email: adriankc@utexas.edu

Content **Page**

Reagents and equipment	3
Plasmid construction	4
**Table S1.** Annotated **P1**-**P2**-**P3**-**P7** sequences	5
**Table S2.** Primers	8
Culturing *E. coli* K207-3 transformed with expression plasmids	9
Polyketide detection and quantification	9
**Figure S1.** Representative high-resolution LC/MS analysis of **1**	10
**Figure S2.** Representative high-resolution LC/MS analysis of **2**	11
**Figure S3.** Representative high-resolution LC/MS analysis of **3**	12
**Figure S4.** Representative high-resolution LC/MS/MS analysis of **1**	13
**Figure S5.** Representative high-resolution LC/MS/MS analysis of **2**	14
**Figure S6.** Representative high-resolution LC/MS/MS analysis of **3**	15
Purification of **1**	16
**Figure S7.** ^{1}H NMR of **1**	16
**Figure S8.** Extracted ion chromatogram calibration curve of **1**	17
**Figure S9.** Calibration curve of lactone UV absorption	17
**Figure S10.** Representative UV absorbance chromatogram and spectrum for **2**	18
**Figure S11.** Representative UV absorbance chromatogram and spectrum for **3**	18
Modeling intermediates in KS substrate tunnels	18
**Table S3.** Key parameters for modeled acyl-KSs	19
**Figure S12.** Interactions between gatekeeping motifs and intermediates	20
**Figure S13.** Possible incompatibility between PikACP3 and AmpKS15	22
References	23

METHODS

Reagents and equipment

All restriction enzymes, HiFi DNA Assembly Master Mix, ligase, and enzyme buffers are from New England Biosciences. Yeast extract, glycerol, sodium chloride, potassium phosphate dibasic, hydrochloric acid, sodium sulfate, ethyl acetate, hexanes, methanol, acetonitrile, and formic acid are from Fisher Scientific. Potassium phosphate monobasic and all primers are from Sigma-Millipore. Sodium propionate is from Alfa Aesar. Isopropyl β-D-1-thiogalactopyranoside (IPTG) is from Carbosynth. Milk filters are from KenAG. Minipreps and gel extractions were performed with Promega Wizard SV kits. Streptomycin and thin layer chromatography (TLC) plates are from Sigma-Aldrich. Luria-Bertani broth is from Fisher Bioreagents. Casein is from Thermo Scientific. Deuterated chloroform is from Camrbidge Isotopes. KAPA polymerase master mix is from Roche Biosciences.

All low-resolution LC/MS as well as the EIC calibration curve for **1** was obtained using an Agilent 6120 system containing a ZORBAX Eclipse Plus C_{18} column (2.1 x 50 mm, 1.8 µm). All high-resolution LC/MS was performed using an Agilent 6230 TOF LC/MS connected to a ZORBAX Eclipse Plus C18 column (2.1 x 50 mm, 1.8 µm). LC/MS/MS data was collected using an Agilent 6530 with a Poroshell 300SB-C3 column (75 x 2.1 mm, 5 µm)

Plasmid construction

The expression plasmid for **P1**-**P2**-**P3**-**P7** was constructed using a BioBricks-like approach that has been described (Table S1)[1]. Briefly, DNA encoding the first and last pikromycin synthase modules, **P1** and **P7**, was inserted into a pCDF-1b backbone, followed by DNA encoding the 2nd and 3rd pikromycin modules, **P2** and **P3**. The DNA encoding each module is split by a T7 terminator, a T7 promoter, a *lac* operator, a ribosomal binding site, and DNA encoding cognate docking domain motifs from the Spinosyn PKS (SpnB/SpnC for **P2**, SpnC/SpnD for **P3**) within a pUC19 cloning plasmid.

DNA encoding the VMYH motif was altered through site-directed mutagenesis (Table S2). For each change, 2 fragments were amplified from the **P3** cloning plasmid using 2 pairs of primers (each consisting of a mutagenic primer that binds the VMYH-encoding region and a primer that binds the pUC19 backbone) and SliCE-assembled[2]. PCRs for the VAYH and VMAH mutations used the **P3** cloning plasmid as a template. PCRs for the VAAH mutation used the **P3(VMAH)** cloning plasmid as the template. Several rounds of mutagenesis were needed to generate the **P3(TNGQ)** cloning plasmid. The original **P3** cloning plasmid was used to generate a **P3(VNGH)** cloning plasmid, which was then used as a template to generate a **P3(TNGH)** cloning plasmid, which was in turn used as a template for the PCR that yielded the final **P3(TNGQ)** cloning plasmid. Since PCR can introduce mutations, each KS-encoded fragment was digested with *MfeI* and *XbaI* and ligated into a **P3** cloning plasmid that had not been PCR-amplified. To make the **P3(AmpKS15)** cloning plasmid, DNA encoding the KS was amplified from *Streptomyces nodosus* ATCC 29757 and SLiCE-assembled into *MfeI*/*XbaI*-digested **P3** cloning plasmid. Mutations and assemblies were checked with overlapping Sanger sequencing reads of the KS-encoding regions.

Each of the **P3** cloning plasmids was digested with *HindIII* and *XbaI* and ligated into the **P1**-**P7** expression plasmid. DNA encoding **P2** was then similarly inserted to generate the expression plasmids encoding the **P1**-**P2**-**P3**-**P7** variants.

P1-P2^{N} on pCDF

MAHHHHHHVGTSSAGITRTGARTPVTGRGAAAWDTGEVRVRRGLPPAGPDHAEHSFSRAPTGDVRAELI
RGEMSTVSKSESEEFVSVSNDAGSAHGTAEPVAVVGISCRVPGARDPREFWELLAAGGQAVTDVPADRW
NAGDFYDPDRSAPGRSNSRWGGFIEDVDRFDAAFFGISPREAAEMDPQQRLALELGWEALERAGIDPSS
LTGTRTGVFAGAIWDDYATLKHRQGGAAITPHTVTGLHRGIIANRLSYTLGLRGPSMVVDSGQSSSLVA
VHLACESLRRGESELALAGGVSLNLVPDSIIGASKFGGLSPDGRAYTFDARANGYVRGEGGGFVVLKRL
SRAVADGDPVLAVIRGSAVNNGGAAQGMTTPDAQAQEAVLREAHERAGTAPADVRYVELHGTGTPVGDP
IEAAALGAALGTGRPAGQPLLVGSVKTNIGHLEGAAGIAGLIKAVLAVRGRALPASLNYETPNPAIPFE
ELNLRVNTEYLPWEPEHDGQRMVVGVSSFGMGGTNAHVVLEEAPGGCRGASVVESTVGGSAVGGGVVPW
VVSAKSAAALDAQIERLAAFASRDRTDGVDAGAVDAGAVDAGAVARVLAGGRAQFEHRAVVVGSGPDDL
AAALAAPEGLVRGVASGVGRVAFVFPGQGTQWAGMGAELLDSSAVFAAAMAECEAALSPYVDWSLEAVV
RQAPGAPTLERVDVVQPVTFAVMVSLARVWQHHGVTPQAVVGHSQGEIAAAYVAGALSLDDAARVVTLR
SKSIAAHLAGKGGMLSLALSEDAVLERLAGFDGLSVAAVNGPTATVVSGDPVQIEELARACEADGVRAR
VIPVDYASHSRQVEIIESELAEVLAGLSPQAPRVPFFSTLEGAWITEPVLDGGYWYRNLRHRVGFAPAV
ETLATDEGFTHFVEVSAHPVLTMALPGTVTGLATLRRDNGGQDRLVASLAEAWANGLAVDWSPLLPSAT
GHHSDLPTYAFQTERHWLGEIEALAPAGEPAVQPAVLRTEAAEPAELDRDEQLRVILDKVRAQTAQVLG
YATGGQIEVDRTFREAGCTSLTGVDLRNRINAAFGVRMAPSMIFDFPTPEALAEQLLLVVHGEAAANPA
GAEPAPVAAAGAVDEPVAIVGMACRLPGGVASPEDLWRLVAGGGDAISEFPQDRGWDVEGLYHPDPEHP
GTSYVRQGGFIENVAGFDAAFFGISPREALAMDPQQRLLLETSWEAVEDAGIDPTSLRGRQVGVFTGAM
THEYGPSLRDGGEGLDGYLLTGNTASVMSGRVSYTLGLEGPALTVDTACSSSLVALHLAVQALRKGEVD
MALAGGVAVMPTPGMFVEFSRQRGLAGDGRSKAFAASADGTSWSEGVGVLLVERLSDARRNGHQVLAVV
RGSA**V**NQDGASNGLTAPNGPSQQRVIRRALADARLTTSDVDVVEAHGTGTRLGDPIEAQALIATYGQGR
DDEQPLRLGSLKSNIGHTQAAAGVSGVIKMVQAMRHGLLPKTLHVDEPSDQIDWSAGAVELLTEAVDWP
EKQDGGLRRAAVSSFGISGTNAHVVLEEAPVVVKLAPAPTSEGASVVEPSVGGSAVGGGVTPWVVSAKS
AAALDAQIERLAAFASRDRTDDADAGAVDAGAVAHVLADGRAQFEHRAVALGAGADDLVQALADPDGLI
RGTASGVGRVAFVFPGQGTQWAGMGAELLDSSAVFAAAMAECEAALSPYVDWSLEAVVRQAPGAPTLER
VDVVQPVTFAVMVSLARVWQHHGVTPQAVVGHSQGEIAAAYVAGALPLDDAARVVTLRSKSIAAHLAGK
GGMLSLALNEDAVLERLSDFDGLSVAAVNGPTATVVSGDPVQIEELAQACKADGFRARIIPVDYASHSR
QVEIIESELAQVLAGLSPQAPRVPFFSTLEGTWITEPVLDGTYWYRNLRHRVGFAPAIETLAVDEGFTH
FVEVSAHPVLTMTLPETVTGLGTLRREQGGQERLVTSLAEAWVNGLPVAWTSLLPATASRPGLPTYAFQ
AERYWLENTPAALATGDDWRYRIDWKRLPAAEGSERTGLSGRWLAVTPEDHSAQAAAVLTALVDAGAKV
EVLTAGADDDREALAARLTALTTGDGFTGVVSLLDGLVPQVAWVQALGDAGIKAPLWSVTQGAVSVGRL
DTPADPDRAMLWGLGRVVALEHPERWAGLVDLPAQPDAAALAHLVTALSGATGEDQIAIRTTGLHARRL
ARAPLHGRRPTRDWQPHGTVLITGGTGALGSHAARWMAHHGAEHLLLVSRSGEQAPGATQLTAELTASG
ARVTIAACDVADPHAMRTLLDAIPAETPLTAVVHTAGALDDGIVDTLTAEQVRRAHRAKAVGASVLDEL
TRDLDLDAFVLFSSVSSTLGIPGQGNYAPHNAYLDALAARRRATGRSAVSVAWGPWDGGGMAAGDGVAE
RLRNHGVPGMDPELALAALESALGRDETAITVADIDWDRFYLAYSSGRPQPLVEELPEVRRIIDARDSA
TSGQGGSSAQGANPLAERLAAAAPGERTEILLGLVRAQAAAVLRMRSPEDVAADRAFKDIGFDSLAGVE
LRNRLTRATGLQLPATLVFDHPTPLALVSLLRSEFLGDEEASAGTFEELDRWAANLPTLARDEATRAQI
TTRLQAILQSLADVSGGTGGGSVPDRLRSATDDELFQLLDNDLELP

P2^{C}**-P3**^{N}

MSNEEKLREYLRRALVDLHQARERLHEAESGEREPIAIVAMSCRYPGDIRSPEDLWRMLSEGG
EGITPFPTDRGWDLDGLYDADPDALGRAYVREGGFLHDAAEFDAEFFGVSPREALAMDPQQRM
LLTTSWEAFERAGIEPASLRGSSTGVFIGLSYQDYAARVPNAPRGVEGYLLTGSTPSVASGRI
AYTFGLEGPATTVDTACSSSLTALHLAVRALRSGECTMALAGGVAMMATPHMFVEFSRQRALA
PDGRSKAFSADADGFGAAEGVGLLLVERLSDARRNGHPVLAVVRGTAVNQDGASNGLTAPNGP
SQQRVIRQALADARLAPGDIDAVETHGTGTSLGDPIEAQGLQATYGKERPAERPLAIGSVKSN
IGHTQAAAGAAGIIKMVLAMRHGTLPKTLHADEPSPHVDWANSGLALVTEPIDWPAGTGPRRA

AVSSFGISGTNAHVVLEQAPDAASSGEVLGADEVPEVSETVAMAGTAGTSEVAEGSEASEAPA
APGSREASLPGHLPWVLSAKDEQSLRGQAAALHAWLSEPAADLSDADGPARLRDVGYTLATSR
TAFAHRAAVTAADRDGFLDGLATLAQGGTSAHVHLDTARDGTTAFLFTGQGSQRPGAGRELYD
RHPVFARALDEICAHLDGHLELPLLDVMFAAEGSAEAALLDETRYTQCALFALEVALFRLVES
WGMRPAALLGHSVGEIAAAHVAGVFSLADAARLVAARGRLMQELPAGGAMLAVQAAEDEIRVW
LETEERYAGRLDVAAVNGPEAAVLSGDADAAREAEAYWSGLGRRTRALRVSHAFHSAHMDGML
DGFRAVLETVEFRRPSLTVVSNVTGLAAGPDDLCDPEYWVRHVRGTVRFLDGVRVLRDLGVRT
CLELGPDGVLTAMAADGLADTPADSAAGSPVGSPAGSPADSAAGALRPRPLLVALLRRKRSET
ETVADALGRAHAHGTGPDWHAWFAGSGAHRVDLPTYSFRRDRYWLDAPAADTAV**N**TAGLGLGT
ADHPLLGAVVSLPDRDGLLLTGRLSLRTHPWLADHAVLGSVLLPGAAMVELAAHAAESAGLRD
VRELTLLEPLVLPEHGGVELRVTVGAPAGEPGGESAGDGARPVSLHSRLADAPAGTAWSCHAT
GLLATDRPELPVAPDRAAMWPPQGAEEVPLDGLYERLDGNGLAFGPLFQGLNAVWRYEGEVFA
DIALPATTNATAPATANGGGSAAAAPYGIHPALLDASLHAIAVGGLVDEPELVRVPFHWSGVT
VHAAGAAAARVRLASAGTDAVSLSLTDGEGRPLVSVERLTLRPVTADQAAASRVGGLMHRVAW
RPYALASSGEQDPHATSYGPTAVLGKDELKVAAALESAGVEVGLYPDLAALSQDVAAGAPAPR
TVLAPLPAGPADGGAEGVRGTVARTLELLQAWLADEHLAGTRLLLVTRGAVRDPEGSGADDGG
EDLSHAAAWGLVRTAQTENPGRFGLLDLADDASSYRTLPSVLSDAGLRDEPQLALHDGTIRLA
RLASVRPETGTAAPALAPEGTVLLTGGTGGLGGLVARHVVGEWGVRRLLLVSRRGTDAPGADE
LVHELEALGADVSVAACDVADREALTAVLDAIPAEHPLTAVVHTAGVLSDGTLPSMTTEDVEH
VLRPKVDAAFLLDELTSTPAYDLAAFVMFSSAAAVFGGAGQGAYAAANATLDALAWRRRAAGL
PALSLGWGLWAETSGMTGELGQADLRRMSRAGIGGISDAEGIALLDAALRDDRHPVLLPLRLD
AAGLRDAAGNDPAGIPALFRDVVGARTVRARPSAASASTTAGTAGTPGTADGAAETAAVTLAD
RAATVDGPARQRLLLEFVVGEVAEVLGHARGHRIDAERGFLDLGFDSLTAVELRNRLNSAGGL
ALPATLVFDHPSPAALASHLDAELPRGASASTVDSALAELDRIEQQLSMLTGEARARDRIATR
LRALHEKWNSAAEVPTGADVLSTLDSATHDEIFEFIDNELDLS

P3^{C}–P7

MANEEKLFGYLKKVTADLHQTRQRLLAAESRSQEPIAIVGMACRLPGGVASPEDLWRLVAGGE
DAISEFPQDRGWDVEGLYDPNPEATGKSYAREAGFLYEAGEFDADFFGISPREALAMDPQQRL
LLEASWEAFEHAGIPAATARGTSVGVFTG**VMYH**DYATRLTDVPEGIEGYLGTGNSGSVASGRV
AYTLGLEGPAVTVDTACSSSLVALHLAVQALRKGEVDMALAGGVTVMSTPSTFVEFSRQRGLA
PDGRSKSFSSTADGTSWSEGVGVLLVERLSDARRKGHRILAVVRGTAVNQDGASSGLTAPNGP
SQQRVIRRALADARLTTSDVDVVEAHGTGTRLGDPIEAQAVIATYGQGRDGEQPLRLGSLKSN
IGHTQAAAGVSGVIKMVQAMRHGVLPKTLHVEKPTDQVDWSAGAVELLTEAMDWPDKGDGGLR
RAAVSSFGVSGTNAHVVLEEAPAAESSPAVEPPAGGGVVPWPVSAKTSAALDAQIGQLAAYAE
DRTDVDPAVAARALVDSRTAMEHRAVAVGDSREALRDALRMPEGLVRGTVTDPGRVAFVFPGQ
GTQWAGMGAELLDSSPEFAAAMAECETALSPYVDWSLEAVVRQAPSAPTLDRVDVVQPVTFAV
MVSLAKVWQHHGITPEAVIGHSQGEIAAAYVAGALTLDDAARVVTLRSKSIAAHLAGKGGMIS
LALSEEATRQRIENLHGLSIAAVNGPTATVVSGDPTQIQELAQACEADGIRARIIPVDYASHS
AHVETIENELADVLAGLSPQTPQVPFFSTLEGTWITEPALDGGYWYRNLRHRVGFAPAVETLA
TDEGFTHFIEVSAHPVLTMTLPDKVTGLATLRREDGGQHRLTTSLAEAWANGLALDWASLLPA
TGALSPAVPDLPTYAFQHRSYWISPAGPGEAPAHTASGREAVAETGLAWGPGAEDLDEEGRRS
AVLAMVMRQAASVLRCDSPEEVPVDRPLREIGFDSLTAVDFRNRVNRLTGLQLPPTVVF**E**HPT
PVALAERISDELAERNWAVAEPSDHEQAEEEKAAAPAGARSGADTGAGAGMFRALFRQAVEDD
RYGEFLDVLAEASAFRPQFASPEACSERLDPVLLAGGPTDRAEGRAVLVGCTGTAANGGPHEF
LRLSTSFQEERDFLAVPLPGYGTGTGTGTALLPADLDTALDAQARAILRAAGDAPVVLLGHSG
GALLAHELAFRLERAHGAPPAGIVLVDPYPPGHQEPIEVWSRQLGEGLFAGELEPMSDARLLA

MGRYARFLAGPRPGRSSAPVLLVRASEPLGDWQEERGDWRAHWDLPHTVADVPGDHFTMMRDH<br>APAVAEAVLSWLDAIEGIEGAGK
**AmpKS15**<br><br>EPIAVVGMGCRFPGGVDSPQALWEMVAGGTDVISEFPDDRGWDLEALRTSGIDDRDTSVSQRG<br>GFLDSIADFDPGFFGISPREAVTMDPQQRLLLETAWEAIERARIDATRLRGTRTGTFIG**TNGQ**<br>DYAYLLVRSLDDATGDVGTGIAASAVSGRLSYTFGLEGPAITVDTACSSSLVALHLAVQSLRN<br>GECTLALAGGVNVMSTPGSLVEFSRQGGLAGDGRCKAFSDSADGTGWSEGAAVLALERLSDAQ<br>RNGHPVLAVIRGSAVNQDGASNGFTAPNGPSQQRVIRQALSNAGLNPADVDVVEAHGTGTPLG<br>DPIEAQSILATYGQDREQPLLLGSIKSNIGHTQSAASGVAGIMKMIMAMRNEVLPKTLHVDRP<br>STHVDWTAGKVELLTENRPWPTAPDRPRRSGVSSFGVSGTNAHVIVEQAPQTP

Table S1. Annotated **P1-P2-P3-P7** sequences. Modules **P1**, **P2**, **P3**, and **P7** are respectively shown in red, orange, yellow, and magenta text (colored as in Figures 1 and 2). Gatekeeping motifs, VMYH and TNGQ, are shown in bolded cyan text. Residues that deviate from the published sequence are shown in bold green text. Sequence encoding a His_6 tag on the first polypeptide is shown in black. Docking motifs from the spinosyn PKS are underlined[1].

**P3** general F	CGCGTTCCTCTTCACCGGCCAGGGCAGTCA
**P3** general R	TGGCCGGTGAAGAGGAACGCGGTGGTGCCGT
**P3(VMAH)**	
VMAH F	ACCGGCGTGATGgccCACGACTACGCCA
VMAH R	TGGCGTAGTCGTGggcCATCACGCCGGT
**P3(VAYH)**	
VAYH F	GCGTCTTCACCGGCGTGgcgTACCACGACTA
VAYH R	TAGTCGTGGTAcgcCACGCCGGTGAAGACGC
**P3(VAAH)**	
VAAH F	TTCACCGGCGTGgcggccCACGACTA
VAAH R	TAGTCGTGggccgcCACGCCGGTGAA
**P3(VNYH)**	
VNYH F	GTCTTCACCGGCGTGaacTACCACGACTACGCCA
VNYH R	TGGCGTAGTCGTGGTAgttCACGCCGGTGAAGAC
**P3(VNGH)**	
VNGH F	CTTCACCGGCGTGaacggtCACGACTACGCCA
VNGH R	TGGCGTAGTCGTGaccgttCACGCCGGTGAAG
**P3(TNGH)**	
TNGH F	CACCGGCacgaacggtCACGACTACGCCA
TNGH R	TGGCGTAGTCGTGaccgttcgtGCCGGTG
**P3(TNGQ)**	
TNGQ F	CGGCacgaacggtcaaGACTACGCCACCCGT
TNGQ R	ACGGGTGGCGTAGTCttgaccgttcgtGCCG
**P3(AmpKS15)**	
AmpKS15 F	GGGAGAGCGGGAACCAATTGCCGTGGTCGGCATGGGCTGCCGCTT
AmpKS15 R	GTACCCGGGGATCCTCTAGAGGGAGTCTGAGGCGCCTGCTCGACGATGA

Table S2. Sequences of primers used to construct **P1**-**P2**-**P3**-**P7** variants. Mutagenic regions appear in lowercase, while homologous regions for SLiCE assembly are underlined.

Culturing *E. coli* K207-3 transformed with expression plasmids

Production of **1**, **2**, and **3** was assessed from media extracts of *E. coli* K207-3 cultures[3]. After transforming an expression plasmid into *E. coli* K207-3, a single colony was used to inoculate 3 mL of LB media containing 100 µg/mL streptomycin. This starter culture was incubated at 37 °C and 240 rpm for 16 h before 400 µL was transferred to 40 mL of expression media [5 g L^{-1} yeast extract, 10 g L^{-1} casein, 15 g L^{-1} glycerol, 10 g L^{-1} NaCl, and 100 mM potassium phosphate buffer (pH 7.6)] containing 100 µg/mL streptomycin in a 250 mL Erlenmeyer flask covered with a milk filter disk. Cells were cultured at 37 °C, 240 rpm until OD_{600} = 0.4. The cultures were cooled to 19 °C, and sodium propionate and IPTG were added to final concentrations of 20 mM and 0.1 mM, respectively. Cultures were incubated at 19 °C and 240 rpm for 7 d.

Polyketide detection and quantification

After 7 d, 0.5 mL samples of culture broth were acidified with 10 µL of aqueous concentrated HCl. The acidified samples were twice extracted with 0.5 mL of ethyl acetate, concentrated *in vacuo*, and resuspended in 500 µL of 1:1 (v/v) methanol/water. High-resolution LC/MS was used to confirm compounds observed by low-resolution LC/MS (Figure S1). 5 µL of the resuspended samples were injected into an Agilent 6230 TOF LC/MS with a ZORBAX Eclipse Plus C_{18} column (2.1 x 50 mm, 1.8 µm) using a flow rate of 1 mL min^{-1} with a gradient of 5% solvent A [water with 0.1% (v/v) formic acid], 95% solvent B [acetonitrile with 0.1% (v/v) formic acid] to 100% solvent B over 12 min followed by 100% solvent B for 3 min. As **1** was only detected at lower gas temperatures, all samples were run with a gas temperature of 200 °C at 150 V.

The Agilent 6230 TOF LC/MS was equipped with a UV detector that allowed for the determination of inferred molar concentration based on a calibration curve of a tetraketide lactone produced in high titers from **P1**-**P5**-**P6**-**P7** that allowed it to be easily purified in a previous study (Figures S9–S11, Data File 1)[4]. Samples for UV detection were prepared the same way as high-resolution LC/MS samples, except that they were only resuspended in 125 µL of 50/50 (v/v) methanol/water. The 4x concentration allowed for UV peaks in the linear range of the calibration curve and is accounted for when calculating the inferred concentration. Unmutated **P1**-**P2**-**P3**-**P7** produced enough product for isolation and a calibration curve depending on the extracted ion chromatogram peak area was used to measure titers of **1** (Figure S8). The samples for quantification of **1** were prepared the same way as for high-resolution LC/MS.

LC/MS/MS samples were prepared identically to high-resolution LC/MS samples. LC/MS/MS data was collected using an Agilent 6530 with a Poroshell 300SB-C3 column

(75 x 2.1 mm, 5 µm) using a flow rate of 0.7 mL/min with a gradient of 5% solvent A [water with 0.1% (v/v) formic acid], 95% solvent B [acetonitrile with 0.1% (v/v) formic acid] to 90% solvent B over 12 min followed by 90% solvent B for 4 min. Fragments were generated via collision induced dissociation (CID) at 10 V for **2** and **3** and 20 V for **1** (Figures S4–S6).

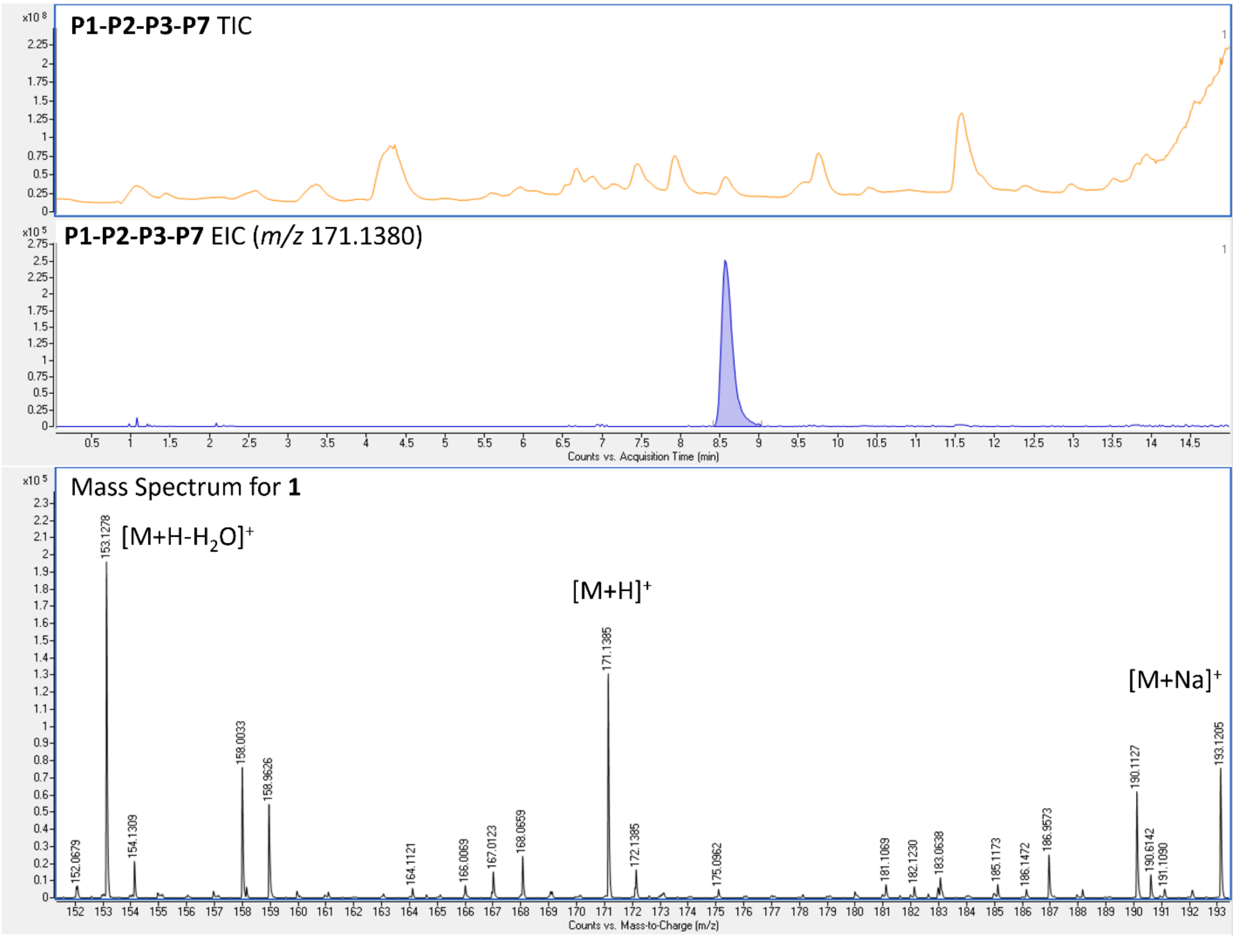

Figure S1. Representative high-resolution LC/MS data of media extract from *E. coli* K207-3 expressing unmutated **P1-P2-P3-P7** with the EIC peak and mass spectrum for **1**. Top, total ion chromatogram. Middle, EIC for 171.1380 *m/z* +/- 100 ppm. Bottom, mass spectrum from the peak at t_R = 8.6 min (**1**, $[M+H]^+$ observed: 171.1385 *m/z*, expected: 171.1380 *m/z*, 3.2 ppm).

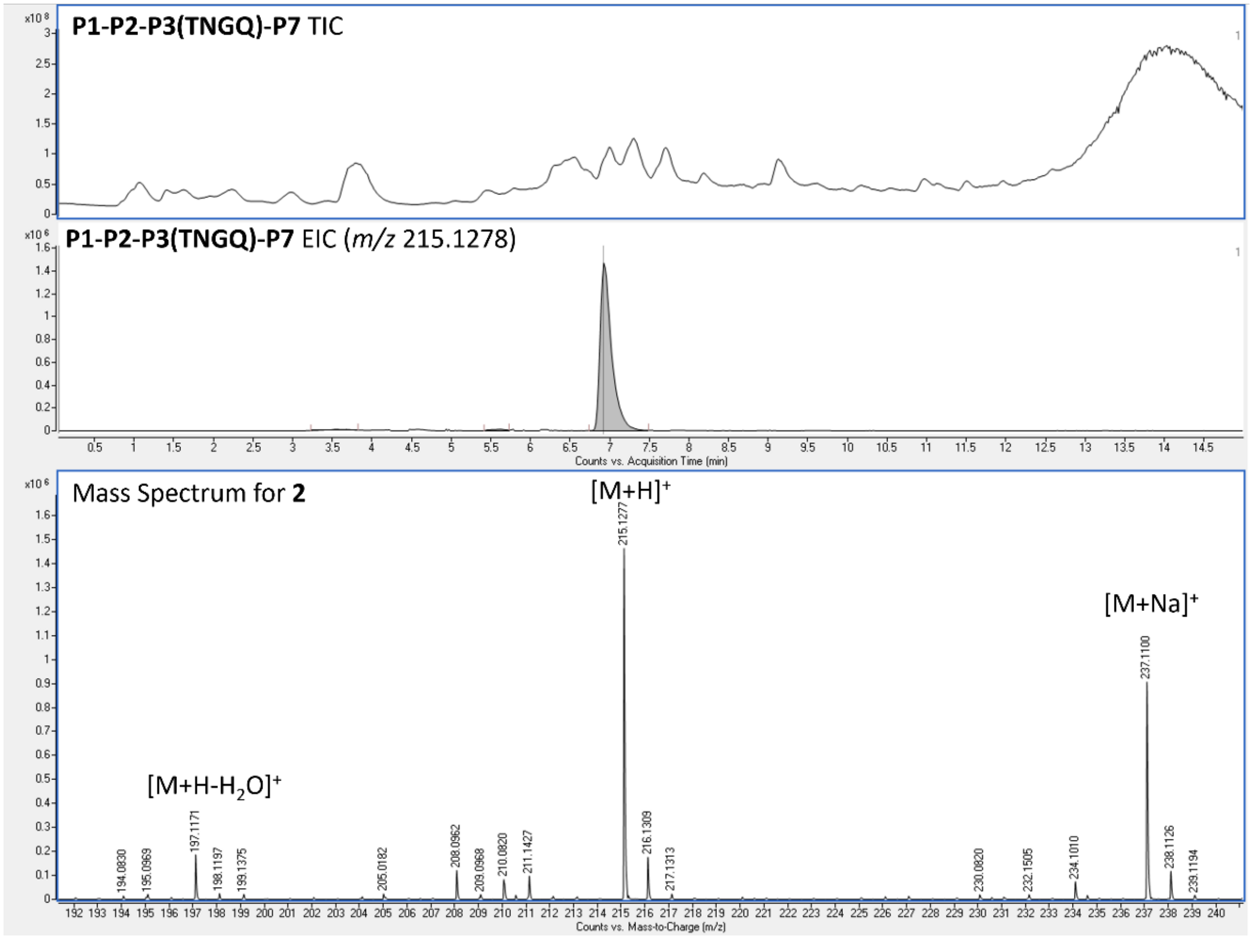

Figure S2. Representative high-resolution LC/MS data of media extract from *E. coli* K207-3 expressing the TNGQ variant of **P1**-**P2**-**P3**-**P7** with the EIC peak and mass spectrum for **2**. Top, total ion chromatogram. Middle, EIC for 215.1277 *m/z* +/- 100 ppm. Bottom, mass spectrum from the peak at t_R = 6.9 min (**2**, $[M+H]^+$ observed: 215.1277 *m/z*, expected: 215.1278 *m/z*, -0.4 ppm).

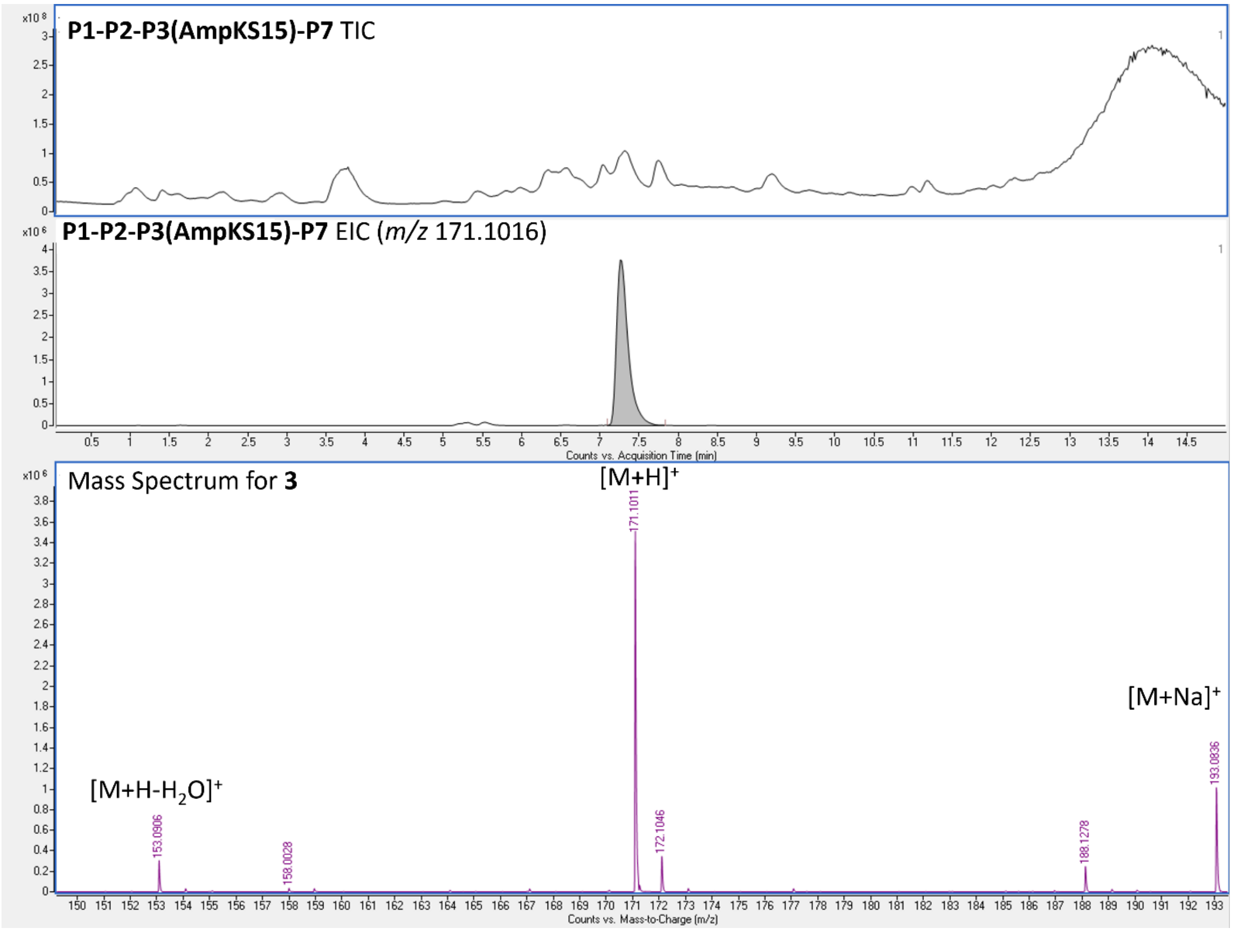

Figure S3. Representative high-resolution LC/MS data of media extract from *E. coli* K207-3 expressing the AmpKS15 variant of **P1**-**P2**-**P3**-**P7** with the EIC peak and mass spectrum for **3**. Top, total ion chromatogram. Middle, EIC for 171.1016 *m/z* +/- 100 ppm. Bottom, mass spectrum from the peak at t_R = 7.4 min (**3**, $[M+H]^+$ observed: 171.1011 *m/z*, expected: 171.1016 *m/z*, -2.8 ppm).

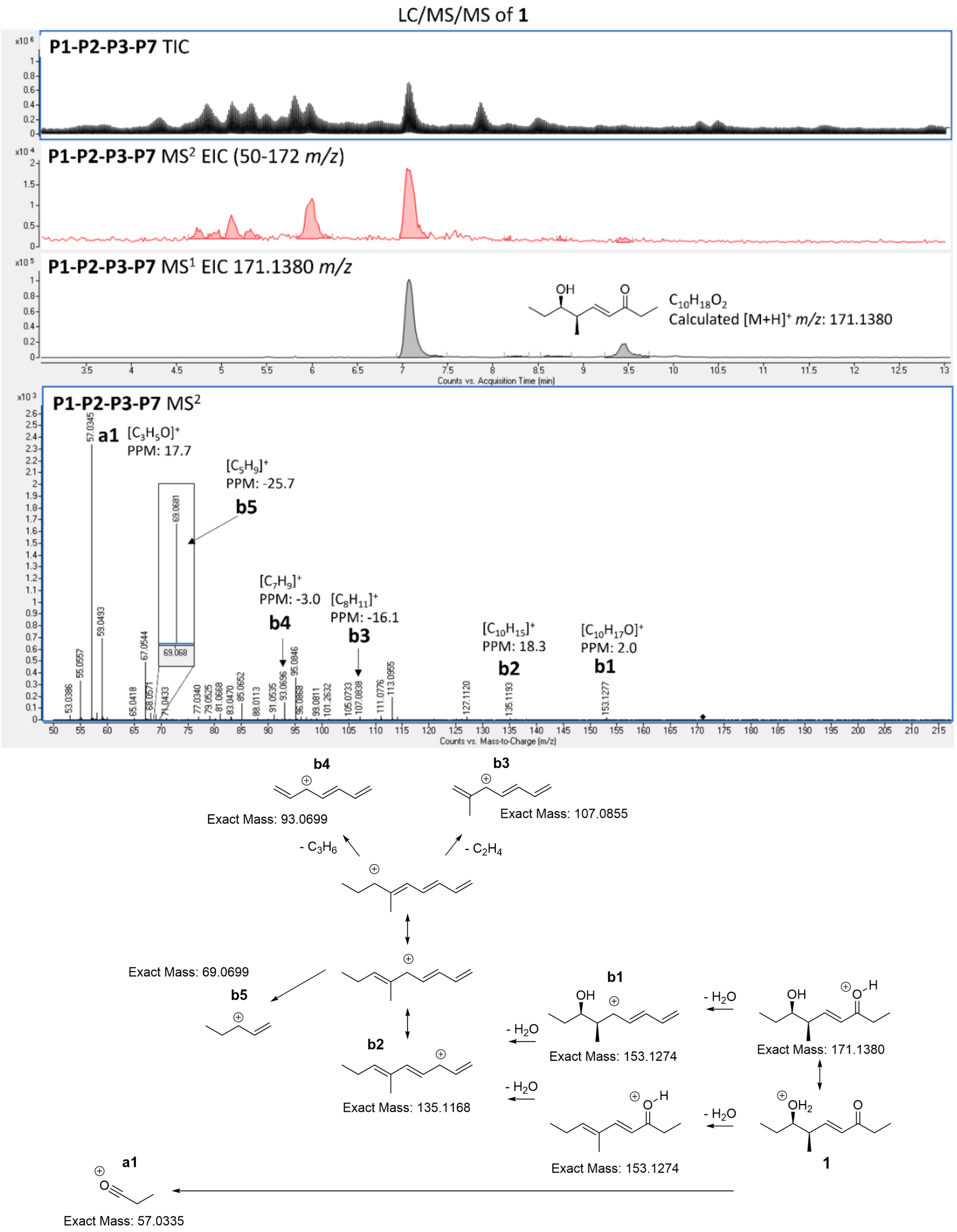

Figure S4. Representative LC/MS/MS data for **1** from *E. coli* K207-3 expressing **P1**-**P2**-**P3**-**P7**. A total ion chromatogram (TIC), an extracted ion chromatogram (EIC) for 50–172 *m/z* in the MS^2 data, and an EIC for 171.1380 *m/z* in the MS^1 data to identify the correct MS^2 EIC peak. The MS^2 spectrum is annotated based on the hypothesized fragmentation pathway (bottom). The integration window for the MS^2 data is 6.99–7.21 min and contains 13 scans. Collision induced dissociation (CID) was performed at 20 V to fragment molecules.

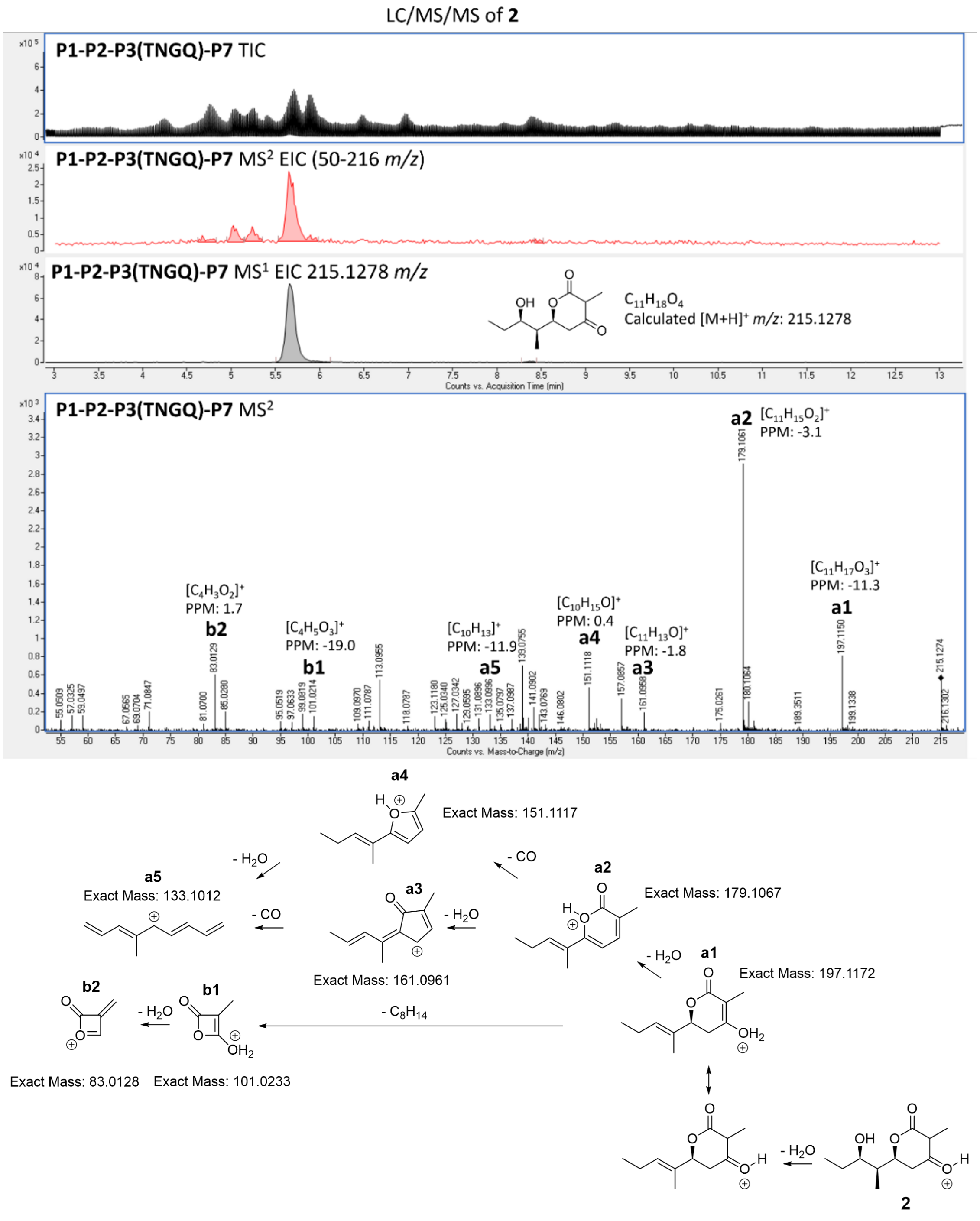

Figure S5. Representative LC/MS/MS data for **2** from *E. coli* K207-3 expressing the TNGQ variant of **P1-P2-P3-P7**. A total ion chromatogram (TIC), an extracted ion chromatogram (EIC) for 50–216 *m/z* in the MS^2 data, and an EIC for 215.1278 *m/z* in the MS^1 data to identify the correct MS^2 EIC peak. The MS^2 spectrum is annotated based on the hypothesized fragmentation pathway (bottom). The integration window for the MS^2 data is 5.65–5.67 min and contains 2 scans. Collision induced dissociation (CID) was performed at 10 V to fragment molecules.

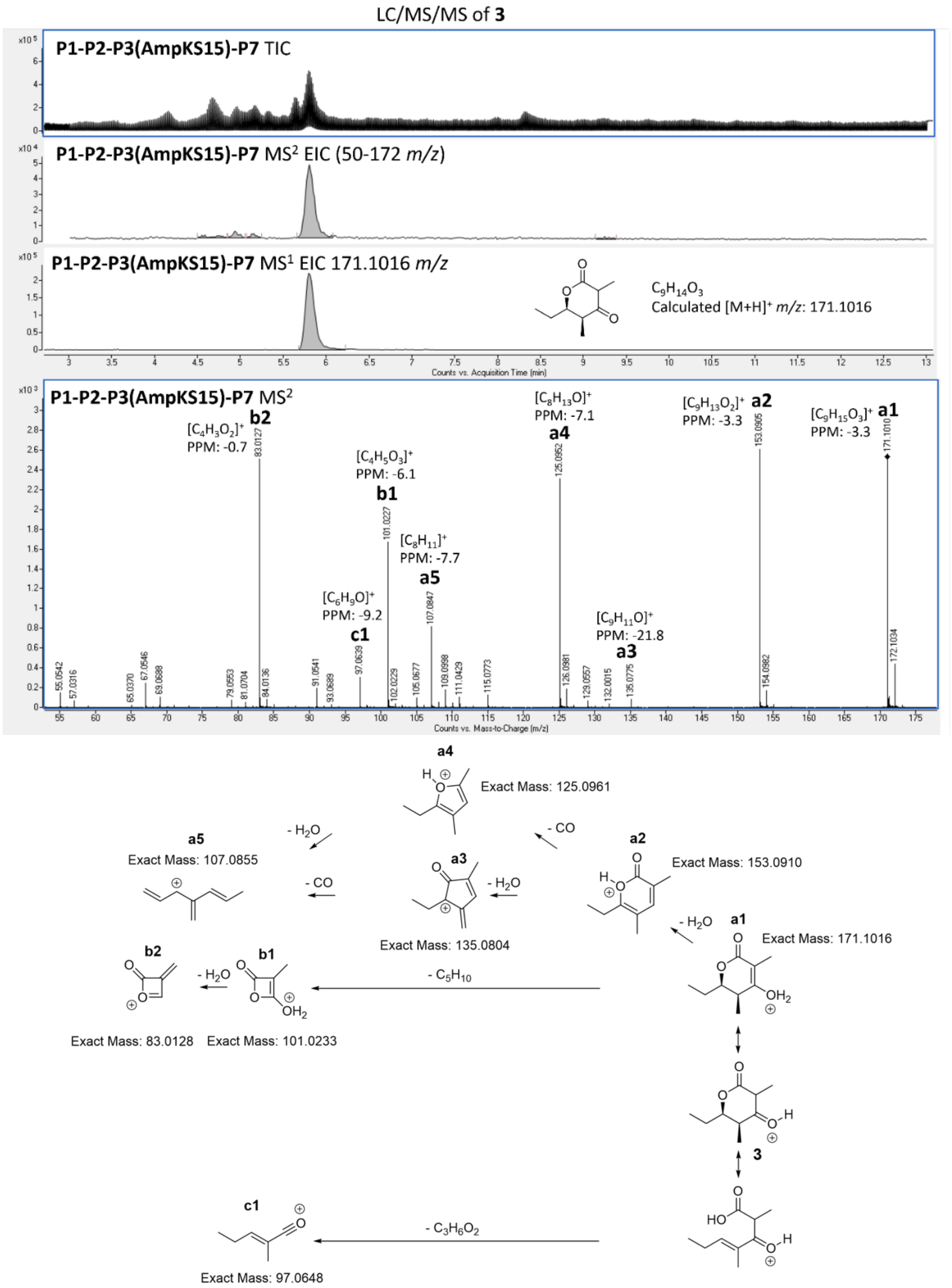

Figure S6. Representative LC/MS/MS data for **3** from *E. coli* K207-3 expressing the AmpKS15 variant of **P1-P2-P3-P7**. A total ion chromatogram (TIC), an extracted ion chromatogram (EIC) for 50–172 *m/z* in the MS^2 data, and an EIC for 171.1016 *m/z* in the MS^1 data to identify the correct MS^2 EIC peak. The MS^2 spectrum is annotated based on the hypothesized fragmentation pathway (bottom). The integration window for the MS^2 data is 5.73–5.93 min and contains 14 scans. Collision induced dissociation (CID) was performed at 10 V to fragment molecules.

Purification of 1

Product **1** was purified from 1 L of *E. coli* K207-3 culture expressing **P1**-**P2**-**P3**-**P7**. After transforming an expression plasmid into *E. coli* K207-3, a single colony was used to inoculate 3 mL of LB media containing 100 µg/mL streptomycin. This starter culture was incubated at 37 °C and 240 rpm for 16 h before 330 µL was transferred to 330 mL of expression media [5 g L^{-1} yeast extract, 10 g L^{-1} casein, 15 g L^{-1} glycerol, 10 g L^{-1} NaCl, and 100 mM potassium phosphate buffer (pH 7.6)] containing 100 µg/mL streptomycin in a 3 L flask covered with a milk filter disk. Cells were cultured at 37 °C, 240 rpm until OD_{600} = 0.6. The cultures were cooled to 16 °C, and sodium propionate and IPTG were added to final concentrations of 20 mM and 0.1 mM, respectively. Cultures were incubated at 19 °C and 240 rpm for 7 d. After incubation, the cultures were extracted twice with ethyl acetate (2 x 1 L) and dried with Na_2SO_4. The ethyl acetate extract was then filtered and dried *in vacuo* and purified by silica gel column chromatography using a 0–100% gradient of ethyl acetate/hexanes. The purest fractions were dried *in vacuo* and used to characterize **1** by ^{1}H NMR and generate the calibration curve (Figures S7 and S8).

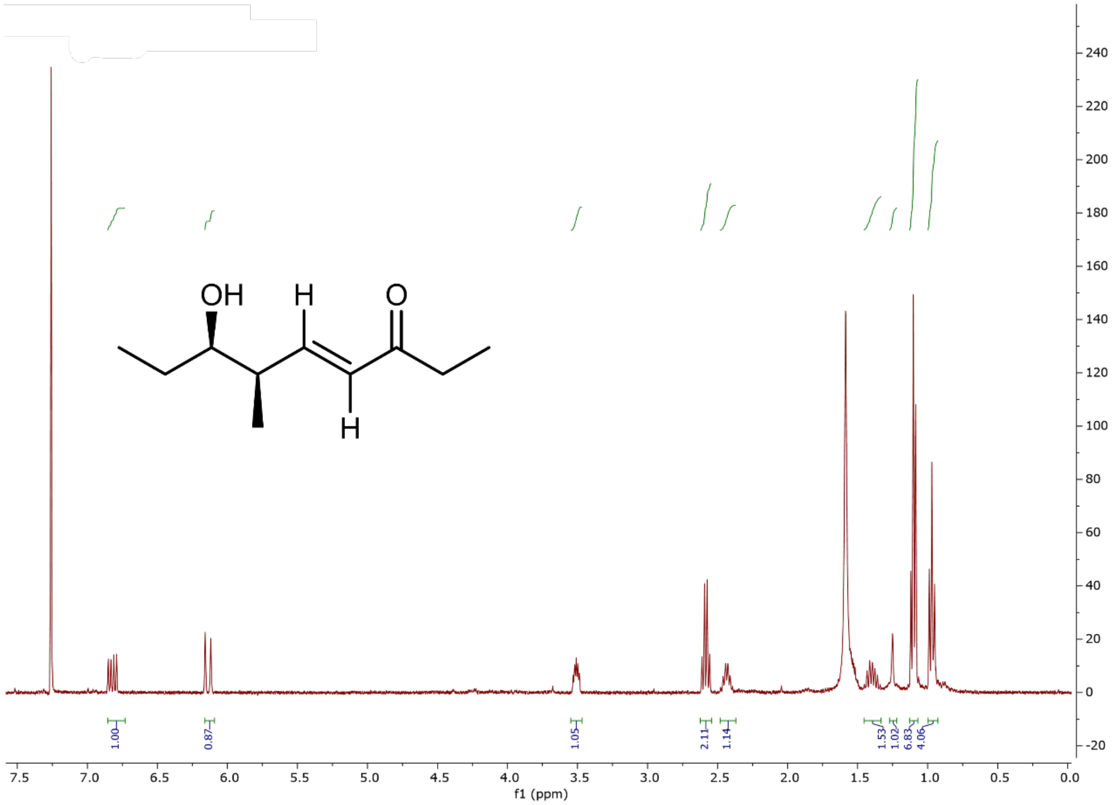

Figure S7. ^{1}H NMR of **1**. ^{1}H NMR (400 MHz, $CDCl_3$) δ 6.82 (dd, *J* = 16.0, 7.8 Hz, 1H), 6.14 (d, *J* = 16.0 Hz, 1H), 3.51 (dt, *J* = 8.9, 4.5 Hz, 1H), 2.58 (q, *J* = 7.3 Hz, 2H), 2.44 (h, *J* = 6.7 Hz, 1H), 1.40 (dp, *J* = 15.0, 7.5 Hz, 1H), 1.14–1.04 (m, 6H), 0.97 (t, *J* = 7.4 Hz, 3H). This spectrum is in agreement with previous characterization[1].

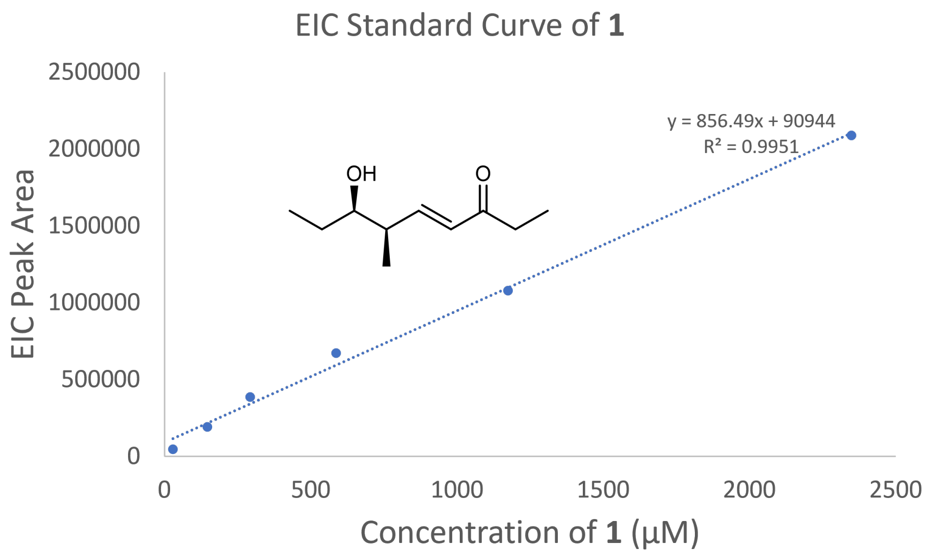

Figure S8. Extracted ion chromatogram calibration curve of **1**. The peak area of the extracted ion chromatogram (EIC) was used for several concentrations of purified **1** to generate a calibration curve.

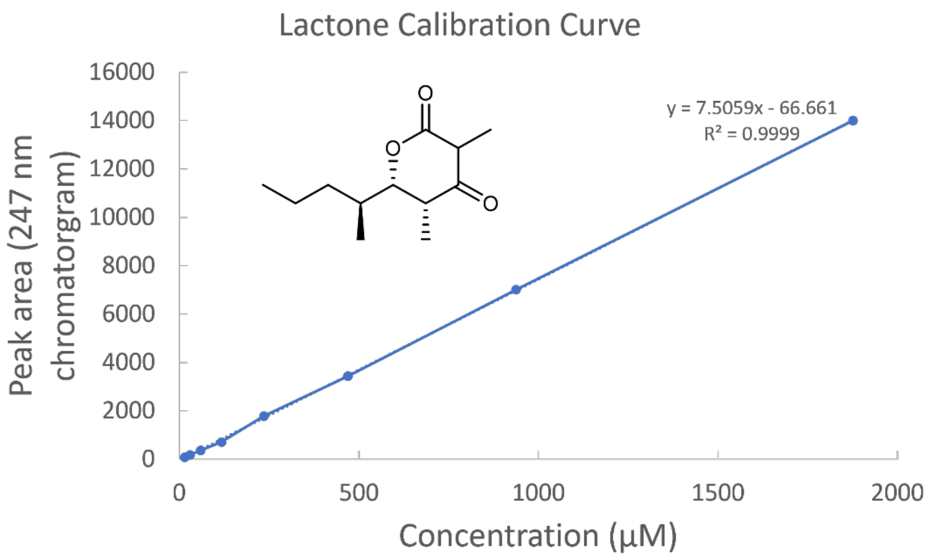

Figure S9. Calibration curve of lactone UV absorption. The absorption of UV light at 247 nm by the β-ketolactone of a reference compound (from **P1**-**P5**-**P6**-**P7**)[4] was used to generate a calibration curve for the quantification of **2** and **3**.

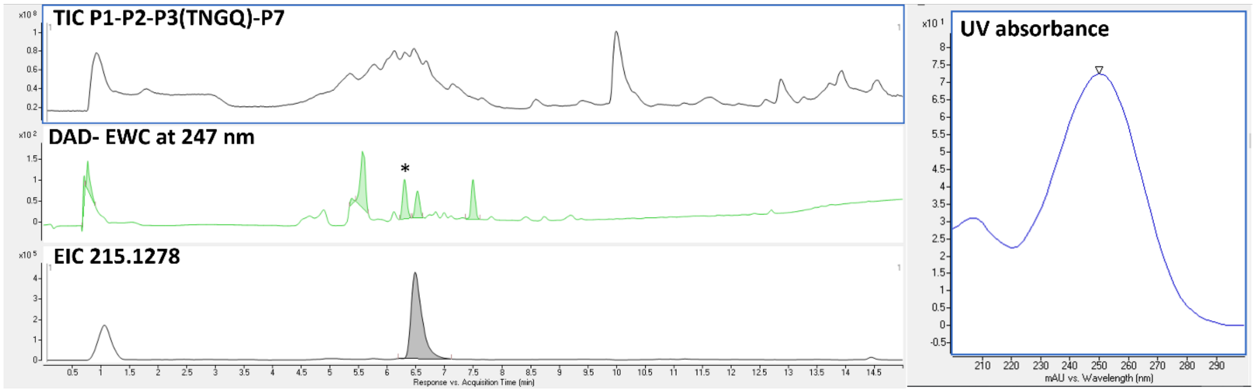

Figure S10. Representative UV absorbance chromatogram and spectrum for **2**. A total ion chromatogram (TIC) for the TNGQ variant of **P1**-**P2**-**P3**-**P7** (top), extracted wavelength chromatogram for 247 nm (EWC, middle), and EIC for **2**. The UV absorbance of the peak at 6.5 min indicated by an asterisk is shown on the right (the delay between the photodiode array and the mass spectrometer is ~10 s).

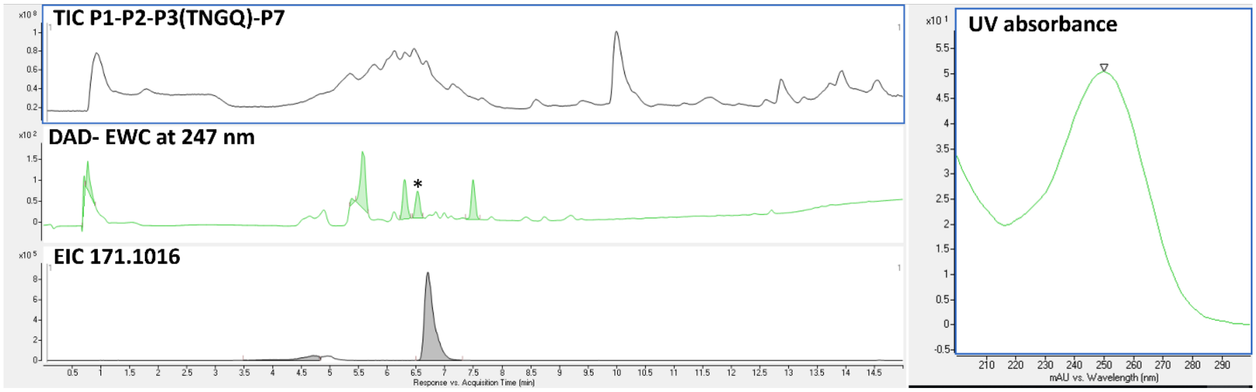

Figure S11. Representative UV absorbance chromatogram and spectrum for **3**. A total ion chromatogram (TIC) for the TNGQ variant of **P1**-**P2**-**P3**-**P7** (top), extracted wavelength chromatogram for 247 nm (EWC, middle), and EIC for **2**. The UV absorbance of the peak at 6.6 min indicated by an asterisk is shown on the right (the delay between the photodiode array and the mass spectrometer is ~10 s).

Modeling intermediates in KS substrate tunnels

The dimeric structures of PikKS3 and its variants as well as AmpKS15 were predicted with AlphaFold 2.0[5], while coordinate and restraint files for the methyl thioester analogs of D-β-D-δ-dihydroxy-L-γ-methylheptanoyl-PikACP3 and D-δ-hydroxy-L-γ-methyl-α-heptenoyl-PikACP3 were generated by the program Sketcher[6] (Figures 4 and S12). The program *Coot* was used to position the intermediates as in experimentally-determined acyl-KS structures (PDBs: 2BUI, 2GFY, 2IX4, 6ROP, 7UK4)[7-13] where the hydrogen bond distance between the thioester oxygen and the amide nitrogen of position 32 ranges from 2.7 to 3.1 Å, the N-C_α-C_β-S torsion angle of the reactive cysteine ranges from -59° to -

96°, and the O-C-C_α-C_β torsion angle of the acyl chain ranges from -48°, to -88° (applicable to the β-hydroxyacyl intermediate, 0° was used for the enoyl intermediate) (Table S3).

Hydrated intermediate bound to KS

KS	O(thioester) – N(32)	N-C_α-C_β-S torsion	O-C-C_α-C_β torsion
PikKS3(VMYH)	2.7 Å	-72.6°	-70.8°
PikKS3(VAYH)	2.7 Å	-73.2°	-73.9°
PikKS3(VMAH)	2.7 Å	-71.7°	-71.1°
PikKS3(VAAH)	2.7 Å	-73.2°	-70.2°
PikKS3(TNGQ)	2.7 Å	-72.4°	-69.5°
AmpKS15	2.7 Å	-68.0°	-72.8°

Dehydrated intermediate bound to KS

KS	O(thioester) – N(32)	N-C_α-C_β-S torsion	O-C-C_α-C_β torsion
PikKS3(VMYH)	3.1 Å	-72.6	0°
PikKS3(VAYH)	3.1 Å	-73.2°	0°
PikKS3(VMAH)	3.1 Å	-71.7°	0°
PikKS3(VAAH)	3.1 Å	-73.2°	0°
PikKS3(TNGQ)	3.1 Å	-72.4°	0°
AmpKS15	3.1 Å	-68.0°	0°

Table S3. Key parameters for modeled acyl-KSs.

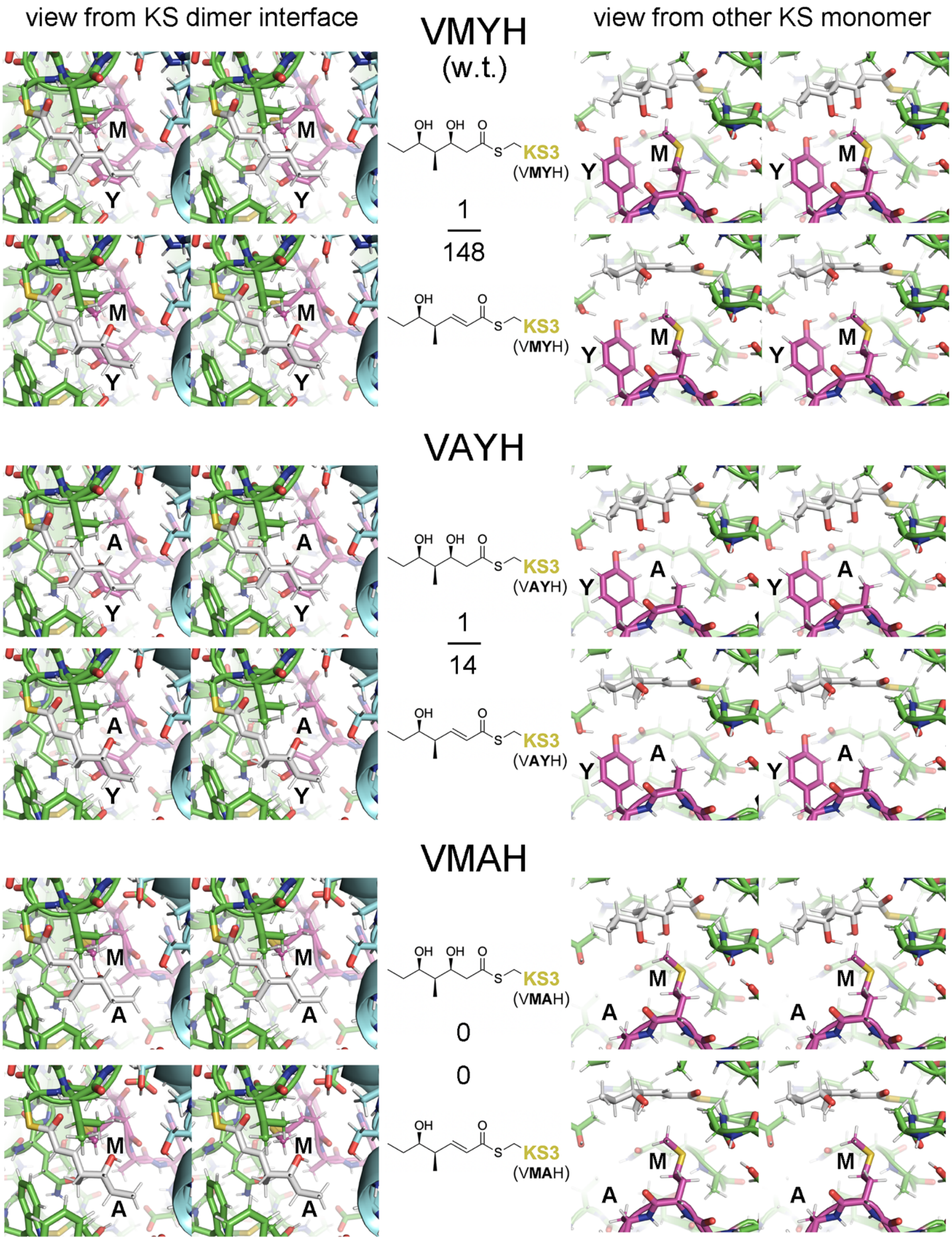

view from KS dimer interface
VMYH
(w.t.)
view from other KS monomer
M
Y
OH
OH
O
S
KS3
(VMYH)
1
148
VAYH
A
(VAYH)
1
14
VMAH
(VMAH)
0
0

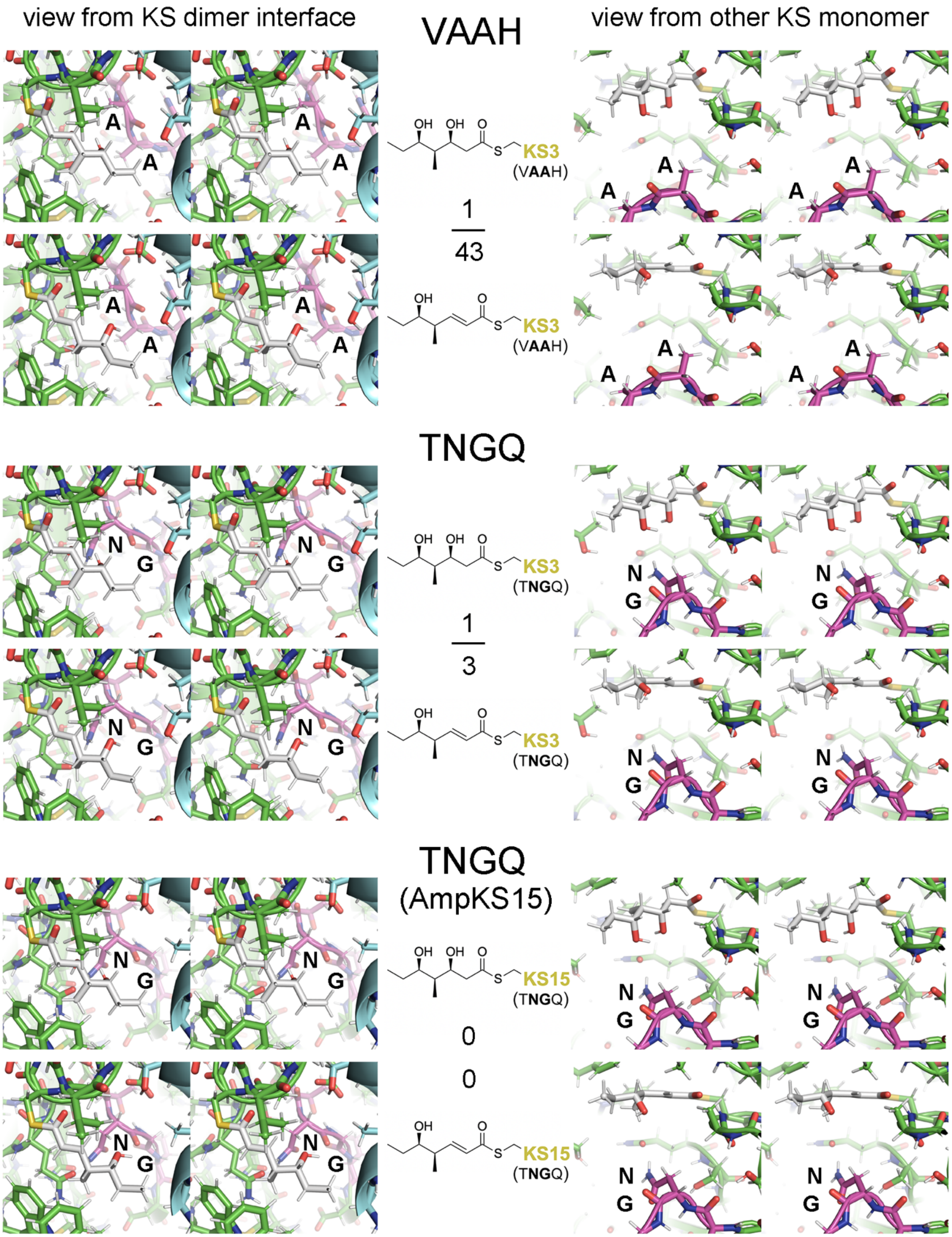

Figure S12. Interactions between gatekeeping residues and intermediates. The hydrated and dehydrated triketide intermediates of **P1**-**P2**-**P3**-**P7** were modeled into PikKS3, its

VAYH, VMAH, VAAH, and TNGQ variants, and AmpKS15. The numbers in the center of each panel are from Figure 3. They report the relative production, or lack thereof, of **1** and **2** by synthases containing the indicated motif and provide a metric for how the β-hydroxy and α,β-unsaturated triketide intermediates pass through PikKS3 and its variants. The stereodiagrams show how the intermediates (grey carbons) may interact with residues in the KS substrate tunnel, especially those in positions 2 and 3 (bold). AmpKS15 is a family F KS that is naturally acylated by a β-hydroxy intermediate and expected to be acylated by the hydrated triketide. Perhaps AmpKS15 is not acylated by the hydrated triketide in the AmpKS15 variant of **P1**-**P2**-**P3**-**P7** variant due to a poor interface with PikACP3 (Figure S13).

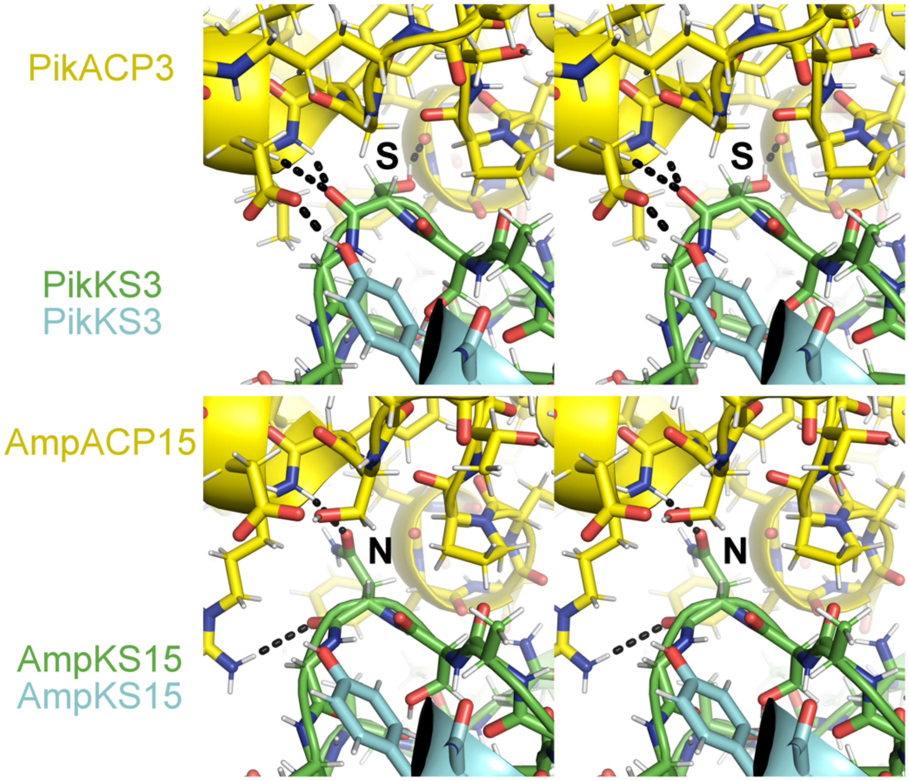

Figure S13. Possible incompatibility between PikACP3 and AmpKS15**.** Stereodiagrams show the Alphafold 2.0 predictions of PikACP3 docking with PikKS3 and AmpACP15 docking with AmpKS15 (ACPs in yellow, KSs in green and cyan). The surface asparagine (**N**) of AmpKS15 is present in >90% of KSs and makes many contacts with AmpACP15 (hydrogen bonds illustrated with dashed lines). PikKS3 contains a less common serine (**S**) at this location that contacts PikACP3 quite differently. Thus, although the dehydrated triketide intermediate shuttled by PikACP3 appears to fit in the AmpKS15 tunnel (Figure S12), it may not enter this tunnel within the AmpKS15 variant of **P1**-**P2**-**P3**-**P7** due to incompatibilities between PikACP3 and AmpKS15.

References

1. K. A. L. Ray, J. D.; Bista, R.; Zhang, J.; Desai, R. R.; Hirsch, M.; Miyazawa, T.; Cordova, A.; Keatinge-Clay, A. T. , *In review at Nat. Commun.*, 2024.
2. K. Motohashi, *Methods Mol Biol*, 2017, **1498**, 349-357.
3. S. Murli, J. Kennedy, L. C. Dayem, J. R. Carney and J. T. Kealey, *J Ind Microbiol Biotechnol*, 2003, **30**, 500-509.
4. J. Zhang, R. Bista, T. Miyazawa and A. T. Keatinge-Clay, *Metab Eng*, 2023, **78**, 93-98.
5. J. Jumper, R. Evans, A. Pritzel, T. Green, M. Figurnov, et al., *Nature*, 2021, **596**, 583-589.
6. J. Agirre, M. Atanasova, H. Bagdonas, C. B. Ballard, A. Basle, et al., *Acta Crystallogr D Struct Biol*, 2023, **79**, 449-461.
7. F. Long, R. A. Nicholls, P. Emsley, S. Graaeulis, A. Merkys, et al., *Acta Crystallogr D Struct Biol*, 2017, **73**, 112-122.
8. P. von Wettstein-Knowles, J. G. Olsen, K. A. McGuire and A. Henriksen, *FEBS J*, 2006, **273**, 695-710.
9. J. Wang, S. M. Soisson, K. Young, W. Shoop, S. Kodali, et al., *Nature*, 2006, **441**, 358-361.
10. C. E. Christensen, B. B. Kragelund, P. von Wettstein-Knowles and A. Henriksen, *Protein Sci*, 2007, **16**, 261-272.
11. A. Rittner, K. S. Paithankar, A. Himmler and M. Grininger, *Protein Sci*, 2020, **29**, 589-605.
12. S. K. Kim, M. S. Dickinson, J. Finer-Moore, Z. Guan, R. M. Kaake, et al., *Nat Struct Mol Biol*, 2023, **30**, 296-308.
13. P. Emsley, B. Lohkamp, W. G. Scott and K. Cowtan, *Acta Crystallogr D Biol Crystallogr*, 2010, **66**, 486-501.
